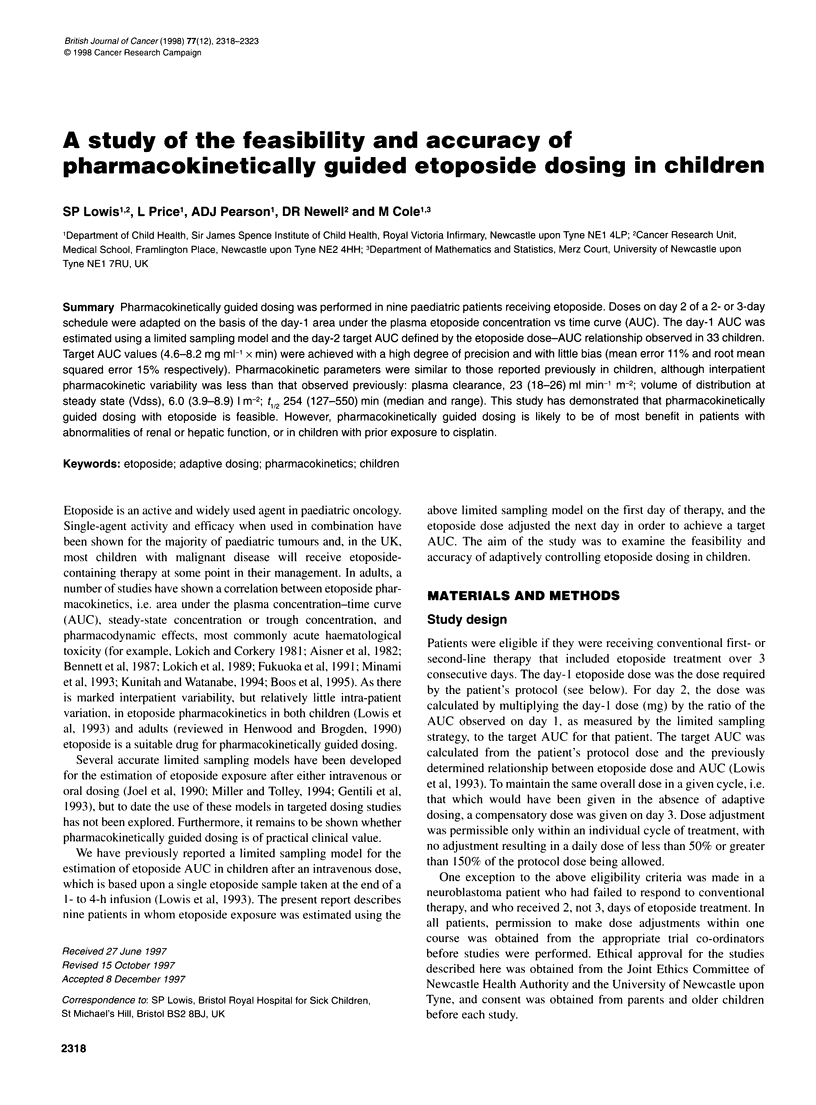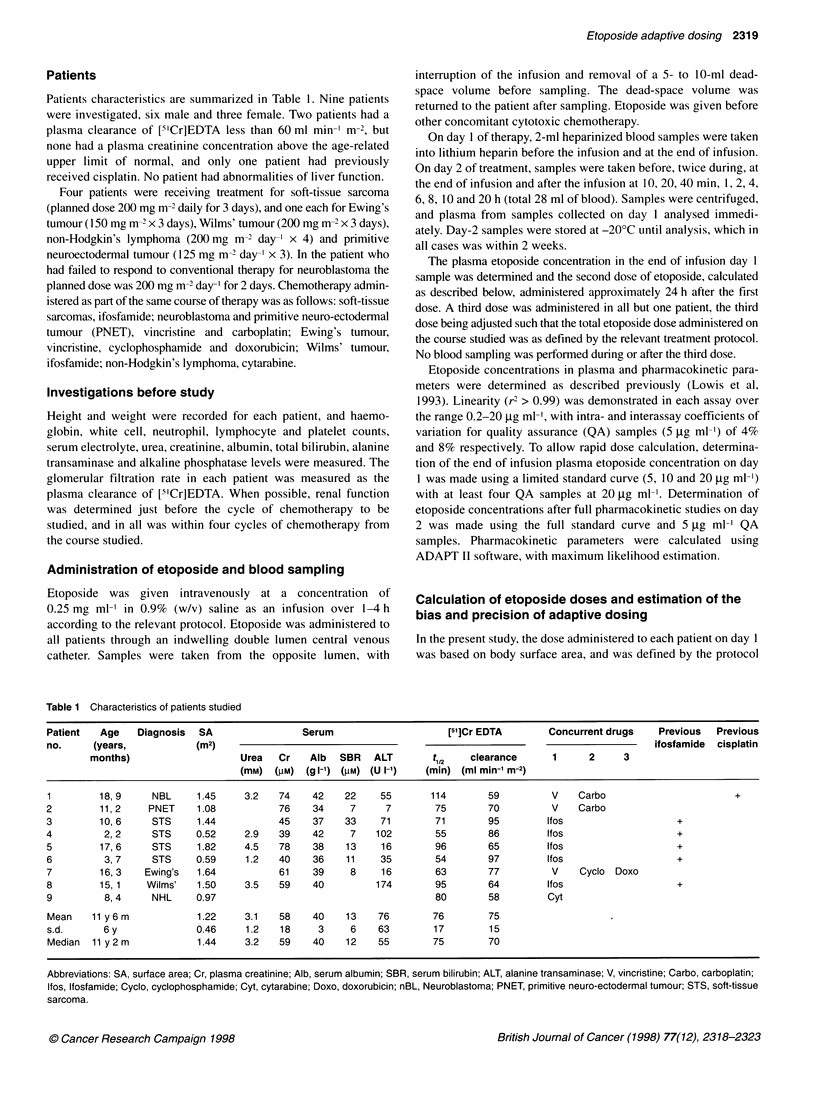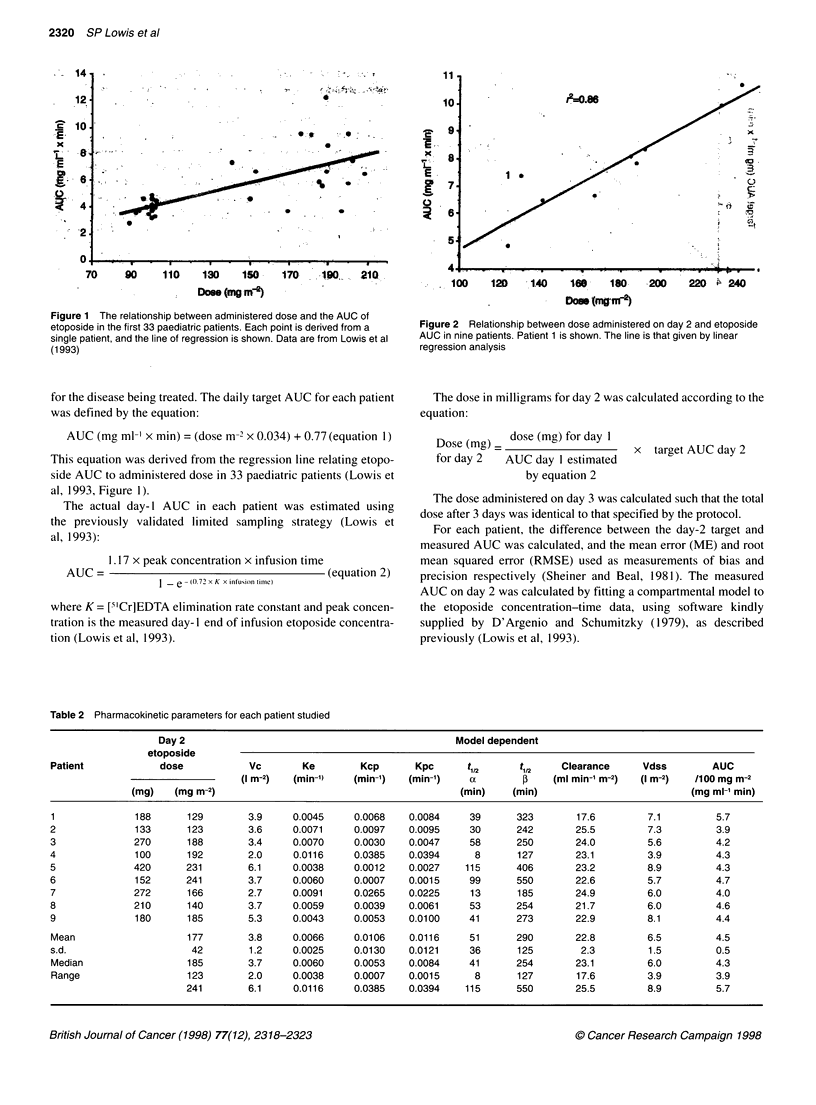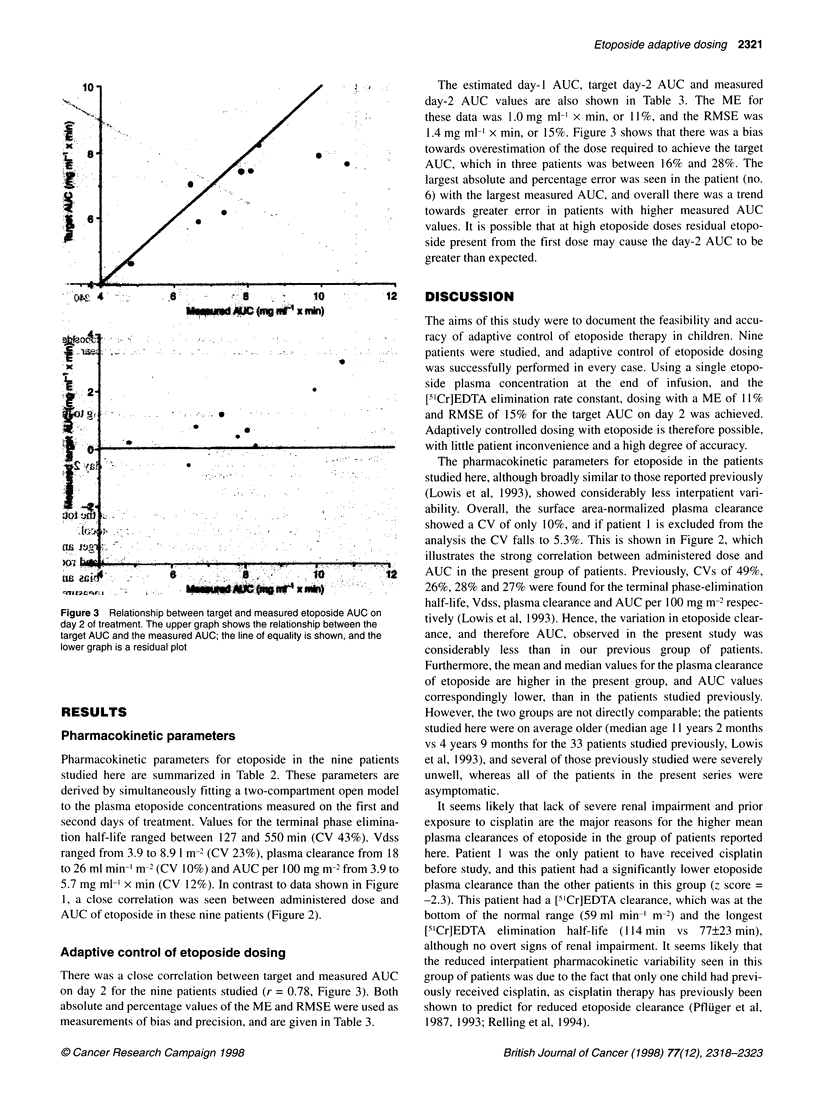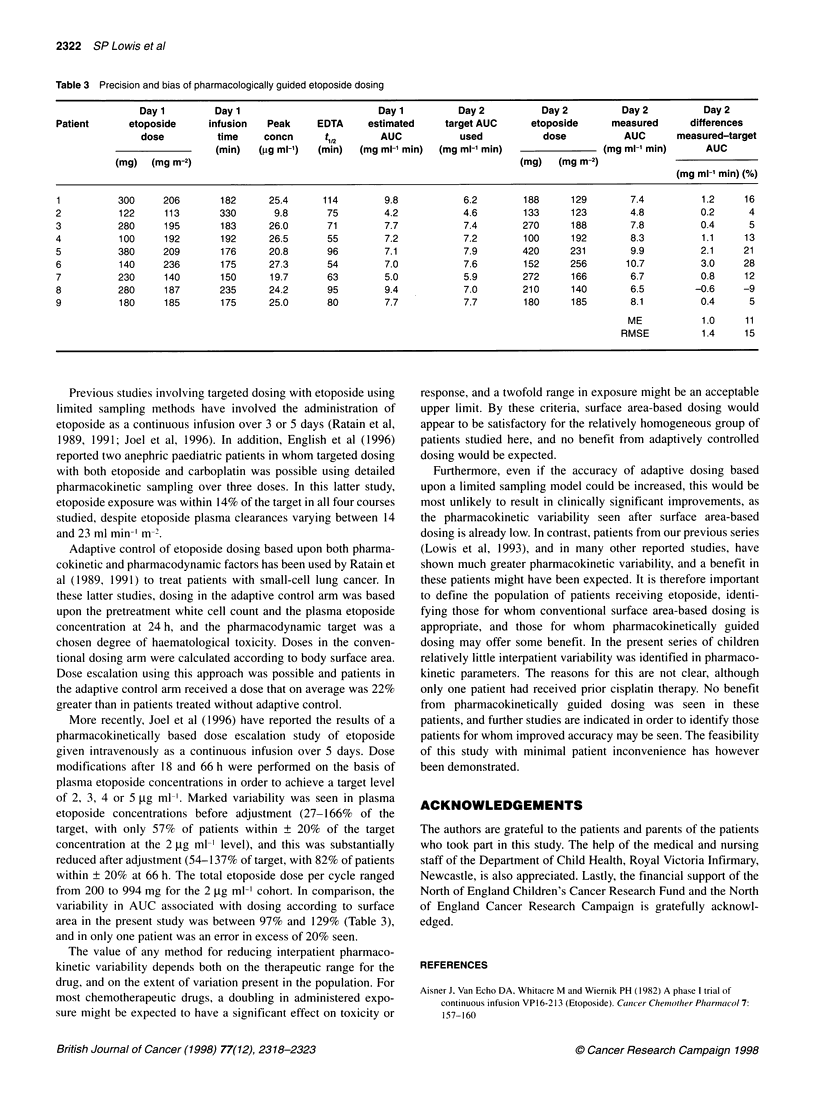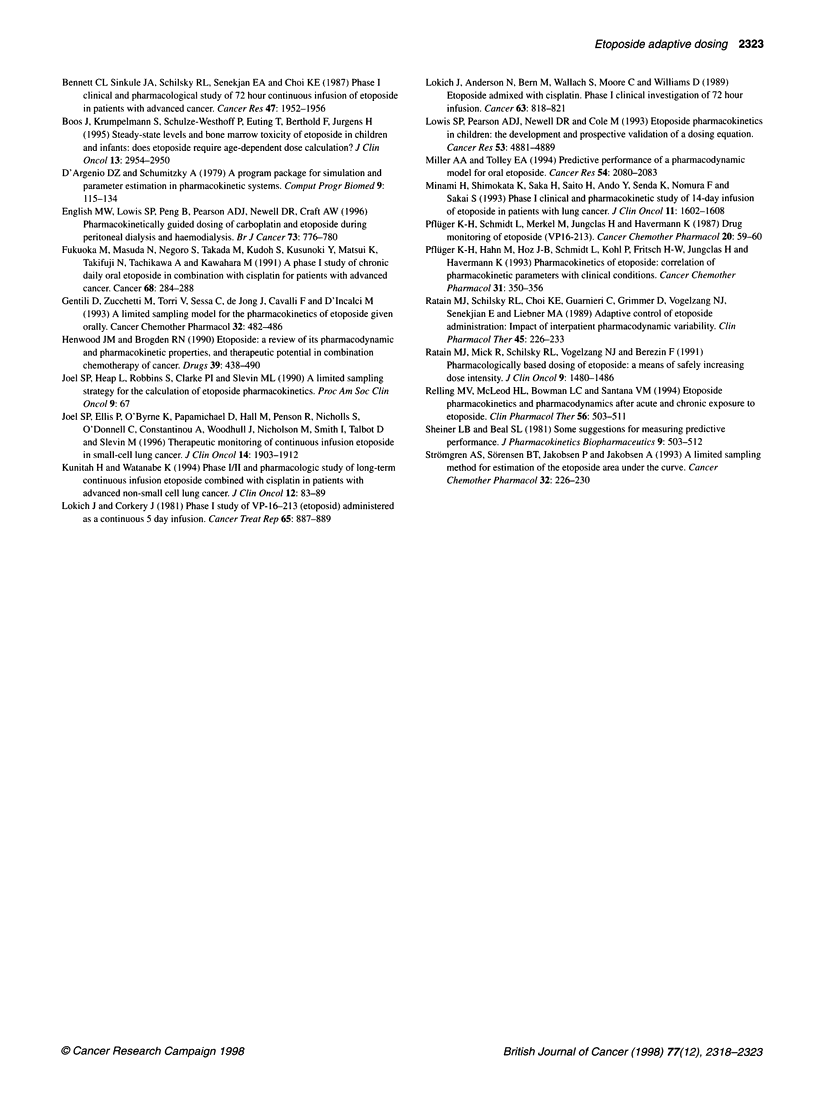# A study of the feasibility and accuracy of pharmacokinetically guided etoposide dosing in children.

**DOI:** 10.1038/bjc.1998.385

**Published:** 1998-06

**Authors:** S. P. Lowis, L. Price, A. D. Pearson, D. R. Newell, M. Cole

**Affiliations:** Department of Child Health, Sir James Spence Institute of Child Health, Royal Victoria Infirmary, Newcastle upon Tyne, UK.

## Abstract

Pharmacokinetically guided dosing was performed in nine paediatric patients receiving etoposide. Doses on day 2 of a 2- or 3-day schedule were adapted on the basis of the day-1 area under the plasma etoposide concentration vs time curve (AUC). The day-1 AUC was estimated using a limited sampling model and the day-2 target AUC defined by the etoposide dose-AUC relationship observed in 33 children. Target AUC values (4.6-8.2 mg ml(-1) x min) were achieved with a high degree of precision and with little bias (mean error 11% and root mean squared error 15% respectively). Pharmacokinetic parameters were similar to those reported previously in children, although interpatient pharmacokinetic variability was less than that observed previously: plasma clearance, 23 (18-26) ml min(-1) m(-2); volume of distribution at steady state (Vdss), 6.0 (3.9-8.9) l m(-2); t(1/2) 254 (127-550) min (median and range). This study has demonstrated that pharmacokinetically guided dosing with etoposide is feasible. However, pharmacokinetically guided dosing is likely to be of most benefit in patients with abnormalities of renal or hepatic function, or in children with prior exposure to cisplatin.


*British Journal of Cancer* (1998) **77**(12), 2318–2323
© 1998 Cancer Research Campaign

### A study of the feasibility and accuracy of pharmacokinetically guided etoposide dosing in children

**SP Lowis[1,2], L Price[1], ADJ Pearson[1], DR Newell[2] and M Cole[1,3]**

[1]Department of Child Health, Sir James Spence Institute of Child Health, Royal Victoria Infirmary, Newcastle upon Tyne NE1 4LP; [2]Cancer Research Unit, Medical School, Framlington Place, Newcastle upon Tyne NE2 4HH; [3]Department of Mathematics and Statistics, Merz Court, University of Newcastle upon Tyne NE1 7RU, UK

**Summary** Pharmacokinetically guided dosing was performed in nine paediatric patients receiving etoposide. Doses on day 2 of a 2- or 3-day schedule were adapted on the basis of the day-1 area under the plasma etoposide concentration vs time curve (AUC). The day-1 AUC was estimated using a limited sampling model and the day-2 target AUC defined by the etoposide dose–AUC relationship observed in 33 children. Target AUC values (4.6–8.2 mg $ml^{-1} \times$ min) were achieved with a high degree of precision and with little bias (mean error 11% and root mean squared error 15% respectively). Pharmacokinetic parameters were similar to those reported previously in children, although interpatient pharmacokinetic variability was less than that observed previously: plasma clearance, 23 (18–26) ml $min^{-1}$ $m^{-2}$; volume of distribution at steady state (Vdss), 6.0 (3.9–8.9) l $m^{-2}$; $t_{1/2}$ 254 (127–550) min (median and range). This study has demonstrated that pharmacokinetically guided dosing with etoposide is feasible. However, pharmacokinetically guided dosing is likely to be of most benefit in patients with abnormalities of renal or hepatic function, or in children with prior exposure to cisplatin.

**Keywords:** etoposide; adaptive dosing; pharmacokinetics; children

Etoposide is an active and widely used agent in paediatric oncology. Single-agent activity and efficacy when used in combination have been shown for the majority of paediatric tumours and, in the UK, most children with malignant disease will receive etoposide-containing therapy at some point in their management. In adults, a number of studies have shown a correlation between etoposide pharmacokinetics, i.e. area under the plasma concentration–time curve (AUC), steady-state concentration or trough concentration, and pharmacodynamic effects, most commonly acute haematological toxicity (for example, Lokich and Corkery 1981; Aisner et al, 1982; Bennett et al, 1987; Lokich et al, 1989; Fukuoka et al, 1991; Minami et al, 1993; Kunitah and Watanabe, 1994; Boos et al, 1995). As there is marked interpatient variability, but relatively little intra-patient variation, in etoposide pharmacokinetics in both children (Lowis et al, 1993) and adults (reviewed in Henwood and Brogden, 1990) etoposide is a suitable drug for pharmacokinetically guided dosing.

Several accurate limited sampling models have been developed for the estimation of etoposide exposure after either intravenous or oral dosing (Joel et al, 1990; Miller and Tolley, 1994; Gentili et al, 1993), but to date the use of these models in targeted dosing studies has not been explored. Furthermore, it remains to be shown whether pharmacokinetically guided dosing is of practical clinical value.

We have previously reported a limited sampling model for the estimation of etoposide AUC in children after an intravenous dose, which is based upon a single etoposide sample taken at the end of a 1- to 4-h infusion (Lowis et al, 1993). The present report describes nine patients in whom etoposide exposure was estimated using the above limited sampling model on the first day of therapy, and the etoposide dose adjusted the next day in order to achieve a target AUC. The aim of the study was to examine the feasibility and accuracy of adaptively controlling etoposide dosing in children.

*Received 27 June 1997*
*Revised 15 October 1997*
*Accepted 8 December 1997*

*Correspondence to*: SP Lowis, Bristol Royal Hospital for Sick Children, St Michael's Hill, Bristol BS2 8BJ, UK

### MATERIALS AND METHODS

### Study design

Patients were eligible if they were receiving conventional first- or second-line therapy that included etoposide treatment over 3 consecutive days. The day-1 etoposide dose was the dose required by the patient's protocol (see below). For day 2, the dose was calculated by multiplying the day-1 dose (mg) by the ratio of the AUC observed on day 1, as measured by the limited sampling strategy, to the target AUC for that patient. The target AUC was calculated from the patient's protocol dose and the previously determined relationship between etoposide dose and AUC (Lowis et al, 1993). To maintain the same overall dose in a given cycle, i.e. that which would have been given in the absence of adaptive dosing, a compensatory dose was given on day 3. Dose adjustment was permissible only within an individual cycle of treatment, with no adjustment resulting in a daily dose of less than 50% or greater than 150% of the protocol dose being allowed.

One exception to the above eligibility criteria was made in a neuroblastoma patient who had failed to respond to conventional therapy, and who received 2, not 3, days of etoposide treatment. In all patients, permission to make dose adjustments within one course was obtained from the appropriate trial co-ordinators before studies were performed. Ethical approval for the studies described here was obtained from the Joint Ethics Committee of Newcastle Health Authority and the University of Newcastle upon Tyne, and consent was obtained from parents and older children before each study.

### Patients

Patients characteristics are summarized in Table 1. Nine patients were investigated, six male and three female. Two patients had a plasma clearance of [$^{51}$Cr]EDTA less than 60 ml min$^{-1}$ m$^{-2}$, but none had a plasma creatinine concentration above the age-related upper limit of normal, and only one patient had previously received cisplatin. No patient had abnormalities of liver function.

Four patients were receiving treatment for soft-tissue sarcoma (planned dose 200 mg m$^{-2}$ daily for 3 days), and one each for Ewing's tumour (150 mg m$^{-2}$ × 3 days), Wilms' tumour (200 mg m$^{-2}$ × 3 days), non-Hodgkin's lymphoma (200 mg m$^{-2}$ day$^{-1}$ × 4) and primitive neuroectodermal tumour (125 mg m$^{-2}$ day$^{-1}$ × 3). In the patient who had failed to respond to conventional therapy for neuroblastoma the planned dose was 200 mg m$^{-2}$ day$^{-1}$ for 2 days. Chemotherapy administered as part of the same course of therapy was as follows: soft-tissue sarcomas, ifosfamide; neuroblastoma and primitive neuro-ectodermal tumour (PNET), vincristine and carboplatin; Ewing's tumour, vincristine, cyclophosphamide and doxorubicin; Wilms' tumour, ifosfamide; non-Hodgkin's lymphoma, cytarabine.

### Investigations before study

Height and weight were recorded for each patient, and haemoglobin, white cell, neutrophil, lymphocyte and platelet counts, serum electrolyte, urea, creatinine, albumin, total bilirubin, alanine transaminase and alkaline phosphatase levels were measured. The glomerular filtration rate in each patient was measured as the plasma clearance of [$^{51}$Cr]EDTA. When possible, renal function was determined just before the cycle of chemotherapy to be studied, and in all was within four cycles of chemotherapy from the course studied.

### Administration of etoposide and blood sampling

Etoposide was given intravenously at a concentration of 0.25 mg ml$^{-1}$ in 0.9% (w/v) saline as an infusion over 1–4 h according to the relevant protocol. Etoposide was administered to all patients through an indwelling double lumen central venous catheter. Samples were taken from the opposite lumen, with interruption of the infusion and removal of a 5- to 10-ml dead-space volume before sampling. The dead-space volume was returned to the patient after sampling. Etoposide was given before other concomitant cytotoxic chemotherapy.

On day 1 of therapy, 2-ml heparinized blood samples were taken into lithium heparin before the infusion and at the end of infusion. On day 2 of treatment, samples were taken before, twice during, at the end of infusion and after the infusion at 10, 20, 40 min, 1, 2, 4, 6, 8, 10 and 20 h (total 28 ml of blood). Samples were centrifuged, and plasma from samples collected on day 1 analysed immediately. Day-2 samples were stored at –20°C until analysis, which in all cases was within 2 weeks.

The plasma etoposide concentration in the end of infusion day 1 sample was determined and the second dose of etoposide, calculated as described below, administered approximately 24 h after the first dose. A third dose was administered in all but one patient, the third dose being adjusted such that the total etoposide dose administered on the course studied was as defined by the relevant treatment protocol. No blood sampling was performed during or after the third dose.

Etoposide concentrations in plasma and pharmacokinetic parameters were determined as described previously (Lowis et al, 1993). Linearity ($r^2 > 0.99$) was demonstrated in each assay over the range 0.2–20 μg ml$^{-1}$, with intra- and interassay coefficients of variation for quality assurance (QA) samples (5 μg ml$^{-1}$) of 4% and 8% respectively. To allow rapid dose calculation, determination of the end of infusion plasma etoposide concentration on day 1 was made using a limited standard curve (5, 10 and 20 μg ml$^{-1}$) with at least four QA samples at 20 μg ml$^{-1}$. Determination of etoposide concentrations after full pharmacokinetic studies on day 2 was made using the full standard curve and 5 μg ml$^{-1}$ QA samples. Pharmacokinetic parameters were calculated using ADAPT II software, with maximum likelihood estimation.

### Calculation of etoposide doses and estimation of the bias and precision of adaptive dosing

In the present study, the dose administered to each patient on day 1 was based on body surface area, and was defined by the protocol

**Table 1** Characteristics of patients studied

| Patient no. | Age (years, months) | Diagnosis | SA (m$^2$) | Serum Urea (mM) | Serum Cr (μM) | Serum Alb (g l$^{-1}$) | Serum SBR (μM) | Serum ALT (U l$^{-1}$) | [$^{51}$]Cr EDTA $t_{1/2}$ (min) | [$^{51}$]Cr EDTA clearance (ml min$^{-1}$ m$^{-2}$) | Concurrent drugs 1 | Concurrent drugs 2 | Concurrent drugs 3 | Previous ifosfamide | Previous cisplatin |
|---|---|---|---|---|---|---|---|---|---|---|---|---|---|---|---|
| 1 | 18, 9 | NBL | 1.45 | 3.2 | 74 | 42 | 22 | 55 | 114 | 59 | V | Carbo | | | + |
| 2 | 11, 2 | PNET | 1.08 | | 76 | 34 | 7 | 7 | 75 | 70 | V | Carbo | | | |
| 3 | 10, 6 | STS | 1.44 | | 45 | 37 | 33 | 71 | 71 | 95 | Ifos | | | + | |
| 4 | 2, 2 | STS | 0.52 | 2.9 | 39 | 42 | 7 | 102 | 55 | 86 | Ifos | | | + | |
| 5 | 17, 6 | STS | 1.82 | 4.5 | 78 | 38 | 13 | 16 | 96 | 65 | Ifos | | | + | |
| 6 | 3, 7 | STS | 0.59 | 1.2 | 40 | 36 | 11 | 35 | 54 | 97 | Ifos | | | + | |
| 7 | 16, 3 | Ewing's | 1.64 | | 61 | 39 | 8 | 16 | 63 | 77 | V | Cyclo | Doxo | | |
| 8 | 15, 1 | Wilms' | 1.50 | 3.5 | 59 | 40 | | 174 | 95 | 64 | Ifos | | | + | |
| 9 | 8, 4 | NHL | 0.97 | | | | | | 80 | 58 | Cyt | | | | |
| Mean | 11 y 6 m | | 1.22 | 3.1 | 58 | 40 | 13 | 76 | 76 | 75 | | | | | |
| s.d. | 6 y | | 0.46 | 1.2 | 18 | 3 | 6 | 63 | 17 | 15 | | | | | |
| Median | 11 y 2 m | | 1.44 | 3.2 | 59 | 40 | 12 | 55 | 75 | 70 | | | | | |

Abbreviations: SA, surface area; Cr, plasma creatinine; Alb, serum albumin; SBR, serum bilirubin; ALT, alanine transaminase; V, vincristine; Carbo, carboplatin; Ifos, Ifosfamide; Cyclo, cyclophosphamide; Cyt, cytarabine; Doxo, doxorubicin; nBL, Neuroblastoma; PNET, primitive neuro-ectodermal tumour; STS, soft-tissue sarcoma.

© Cancer Research Campaign 1998

**Figure 1** The relationship between administered dose and the AUC of etoposide in the first 33 paediatric patients. Each point is derived from a single patient, and the line of regression is shown. Data are from Lowis et al (1993)

**Figure 2** Relationship between dose administered on day 2 and etoposide AUC in nine patients. Patient 1 is shown. The line is that given by linear regression analysis for the disease being treated. The daily target AUC for each patient was defined by the equation:

$$\text{AUC (mg ml}^{-1} \times \text{min)} = (\text{dose m}^{-2} \times 0.034) + 0.77 \quad \text{(equation 1)}$$

This equation was derived from the regression line relating etoposide AUC to administered dose in 33 paediatric patients (Lowis et al, 1993, Figure 1).

The actual day-1 AUC in each patient was estimated using the previously validated limited sampling strategy (Lowis et al, 1993):

$$\text{AUC} = \frac{1.17 \times \text{peak concentration} \times \text{infusion time}}{1 - e^{-(0.72 \times K \times \text{infusion time})}} \quad \text{(equation 2)}$$

where $K$ = [$^{51}$Cr]EDTA elimination rate constant and peak concentration is the measured day-1 end of infusion etoposide concentration (Lowis et al, 1993).

The dose in milligrams for day 2 was calculated according to the equation:

$$\text{Dose (mg) for day 2} = \frac{\text{dose (mg) for day 1}}{\text{AUC day 1 estimated by equation 2}} \times \text{target AUC day 2}$$

The dose administered on day 3 was calculated such that the total dose after 3 days was identical to that specified by the protocol.

For each patient, the difference between the day-2 target and measured AUC was calculated, and the mean error (ME) and root mean squared error (RMSE) used as measurements of bias and precision respectively (Sheiner and Beal, 1981). The measured AUC on day 2 was calculated by fitting a compartmental model to the etoposide concentration–time data, using software kindly supplied by D'Argenio and Schumitzky (1979), as described previously (Lowis et al, 1993).

**Table 2** Pharmacokinetic parameters for each patient studied

| Patient | Day 2 etoposide dose | | Model dependent | | | | | | | | |
|---|---|---|---|---|---|---|---|---|---|---|---|
| | (mg) | (mg m$^{-2}$) | Vc (l m$^{-2}$) | Ke (min$^{-1}$) | Kcp (min$^{-1}$) | Kpc (min$^{-1}$) | $t_{1/2}$ α (min) | $t_{1/2}$ β (min) | Clearance (ml min$^{-1}$ m$^{-2}$) | Vdss (l m$^{-2}$) | AUC /100 mg m$^{-2}$ (mg ml$^{-1}$ min) |
| 1 | 188 | 129 | 3.9 | 0.0045 | 0.0068 | 0.0084 | 39 | 323 | 17.6 | 7.1 | 5.7 |
| 2 | 133 | 123 | 3.6 | 0.0071 | 0.0097 | 0.0095 | 30 | 242 | 25.5 | 7.3 | 3.9 |
| 3 | 270 | 188 | 3.4 | 0.0070 | 0.0030 | 0.0047 | 58 | 250 | 24.0 | 5.6 | 4.2 |
| 4 | 100 | 192 | 2.0 | 0.0116 | 0.0385 | 0.0394 | 8 | 127 | 23.1 | 3.9 | 4.3 |
| 5 | 420 | 231 | 6.1 | 0.0038 | 0.0012 | 0.0027 | 115 | 406 | 23.2 | 8.9 | 4.3 |
| 6 | 152 | 241 | 3.7 | 0.0060 | 0.0007 | 0.0015 | 99 | 550 | 22.6 | 5.7 | 4.7 |
| 7 | 272 | 166 | 2.7 | 0.0091 | 0.0265 | 0.0225 | 13 | 185 | 24.9 | 6.0 | 4.0 |
| 8 | 210 | 140 | 3.7 | 0.0059 | 0.0039 | 0.0061 | 53 | 254 | 21.7 | 6.0 | 4.6 |
| 9 | 180 | 185 | 5.3 | 0.0043 | 0.0053 | 0.0100 | 41 | 273 | 22.9 | 8.1 | 4.4 |
| Mean | | 177 | 3.8 | 0.0066 | 0.0106 | 0.0116 | 51 | 290 | 22.8 | 6.5 | 4.5 |
| s.d. | | 42 | 1.2 | 0.0025 | 0.0130 | 0.0121 | 36 | 125 | 2.3 | 1.5 | 0.5 |
| Median | | 185 | 3.7 | 0.0060 | 0.0053 | 0.0084 | 41 | 254 | 23.1 | 6.0 | 4.3 |
| Range | | 123 | 2.0 | 0.0038 | 0.0007 | 0.0015 | 8 | 127 | 17.6 | 3.9 | 3.9 |
| | | 241 | 6.1 | 0.0116 | 0.0385 | 0.0394 | 115 | 550 | 25.5 | 8.9 | 5.7 |

*© Cancer Research Campaign 1998*

**Figure 3** Relationship between target and measured etoposide AUC on day 2 of treatment. The upper graph shows the relationship between the target AUC and the measured AUC; the line of equality is shown, and the lower graph is a residual plot

### RESULTS

### Pharmacokinetic parameters

Pharmacokinetic parameters for etoposide in the nine patients studied here are summarized in Table 2. These parameters are derived by simultaneously fitting a two-compartment open model to the plasma etoposide concentrations measured on the first and second days of treatment. Values for the terminal phase elimination half-life ranged between 127 and 550 min (CV 43%). Vdss ranged from 3.9 to 8.9 l $m^{-2}$ (CV 23%), plasma clearance from 18 to 26 ml $min^{-1}$ $m^{-2}$ (CV 10%) and AUC per 100 mg $m^{-2}$ from 3.9 to 5.7 mg $ml^{-1} \times$ min (CV 12%). In contrast to data shown in Figure 1, a close correlation was seen between administered dose and AUC of etoposide in these nine patients (Figure 2).

### Adaptive control of etoposide dosing

There was a close correlation between target and measured AUC on day 2 for the nine patients studied ($r$ = 0.78, Figure 3). Both absolute and percentage values of the ME and RMSE were used as measurements of bias and precision, and are given in Table 3.

The estimated day-1 AUC, target day-2 AUC and measured day-2 AUC values are also shown in Table 3. The ME for these data was 1.0 mg $ml^{-1} \times$ min, or 11%, and the RMSE was 1.4 mg $ml^{-1} \times$ min, or 15%. Figure 3 shows that there was a bias towards overestimation of the dose required to achieve the target AUC, which in three patients was between 16% and 28%. The largest absolute and percentage error was seen in the patient (no. 6) with the largest measured AUC, and overall there was a trend towards greater error in patients with higher measured AUC values. It is possible that at high etoposide doses residual etoposide present from the first dose may cause the day-2 AUC to be greater than expected.

### DISCUSSION

The aims of this study were to document the feasibility and accuracy of adaptive control of etoposide therapy in children. Nine patients were studied, and adaptive control of etoposide dosing was successfully performed in every case. Using a single etoposide plasma concentration at the end of infusion, and the [$^{51}$Cr]EDTA elimination rate constant, dosing with a ME of 11% and RMSE of 15% for the target AUC on day 2 was achieved. Adaptively controlled dosing with etoposide is therefore possible, with little patient inconvenience and a high degree of accuracy.

The pharmacokinetic parameters for etoposide in the patients studied here, although broadly similar to those reported previously (Lowis et al, 1993), showed considerably less interpatient variability. Overall, the surface area-normalized plasma clearance showed a CV of only 10%, and if patient 1 is excluded from the analysis the CV falls to 5.3%. This is shown in Figure 2, which illustrates the strong correlation between administered dose and AUC in the present group of patients. Previously, CVs of 49%, 26%, 28% and 27% were found for the terminal phase-elimination half-life, Vdss, plasma clearance and AUC per 100 mg $m^{-2}$ respectively (Lowis et al, 1993). Hence, the variation in etoposide clearance, and therefore AUC, observed in the present study was considerably less than in our previous group of patients. Furthermore, the mean and median values for the plasma clearance of etoposide are higher in the present group, and AUC values correspondingly lower, than in the patients studied previously. However, the two groups are not directly comparable; the patients studied here were on average older (median age 11 years 2 months vs 4 years 9 months for the 33 patients studied previously, Lowis et al, 1993), and several of those previously studied were severely unwell, whereas all of the patients in the present series were asymptomatic.

It seems likely that lack of severe renal impairment and prior exposure to cisplatin are the major reasons for the higher mean plasma clearances of etoposide in the group of patients reported here. Patient 1 was the only patient to have received cisplatin before study, and this patient had a significantly lower etoposide plasma clearance than the other patients in this group ($z$ score = –2.3). This patient had a [$^{51}$Cr]EDTA clearance, which was at the bottom of the normal range (59 ml $min^{-1}$ $m^{-2}$) and the longest [$^{51}$Cr]EDTA elimination half-life (114 min vs 77±23 min), although no overt signs of renal impairment. It seems likely that the reduced interpatient pharmacokinetic variability seen in this group of patients was due to the fact that only one child had previously received cisplatin, as cisplatin therapy has previously been shown to predict for reduced etoposide clearance (Pflüger et al, 1987, 1993; Relling et al, 1994).

© Cancer Research Campaign 1998

**Table 3** Precision and bias of pharmacologically guided etoposide dosing

| Patient | Day 1 etoposide dose (mg) | Day 1 etoposide dose (mg $m^{-2}$) | Day 1 infusion time (min) | Peak concn (μg $ml^{-1}$) | EDTA $t_{1/2}$ (min) | Day 1 estimated AUC (mg $ml^{-1}$ min) | Day 2 target AUC used (mg $ml^{-1}$ min) | Day 2 etoposide dose (mg) | Day 2 etoposide dose (mg $m^{-2}$) | Day 2 measured AUC (mg $ml^{-1}$ min) | Day 2 differences measured–target AUC (mg $ml^{-1}$ min) | Day 2 differences measured–target AUC (%) |
|---|---|---|---|---|---|---|---|---|---|---|---|---|
| 1 | 300 | 206 | 182 | 25.4 | 114 | 9.8 | 6.2 | 188 | 129 | 7.4 | 1.2 | 16 |
| 2 | 122 | 113 | 330 | 9.8 | 75 | 4.2 | 4.6 | 133 | 123 | 4.8 | 0.2 | 4 |
| 3 | 280 | 195 | 183 | 26.0 | 71 | 7.7 | 7.4 | 270 | 188 | 7.8 | 0.4 | 5 |
| 4 | 100 | 192 | 192 | 26.5 | 55 | 7.2 | 7.2 | 100 | 192 | 8.3 | 1.1 | 13 |
| 5 | 380 | 209 | 176 | 20.8 | 96 | 7.1 | 7.9 | 420 | 231 | 9.9 | 2.1 | 21 |
| 6 | 140 | 236 | 175 | 27.3 | 54 | 7.0 | 7.6 | 152 | 256 | 10.7 | 3.0 | 28 |
| 7 | 230 | 140 | 150 | 19.7 | 63 | 5.0 | 5.9 | 272 | 166 | 6.7 | 0.8 | 12 |
| 8 | 280 | 187 | 235 | 24.2 | 95 | 9.4 | 7.0 | 210 | 140 | 6.5 | –0.6 | –9 |
| 9 | 180 | 185 | 175 | 25.0 | 80 | 7.7 | 7.7 | 180 | 185 | 8.1 | 0.4 | 5 |
| | | | | | | | | | | ME | 1.0 | 11 |
| | | | | | | | | | | RMSE | 1.4 | 15 |

Previous studies involving targeted dosing with etoposide using limited sampling methods have involved the administration of etoposide as a continuous infusion over 3 or 5 days (Ratain et al, 1989, 1991; Joel et al, 1996). In addition, English et al (1996) reported two anephric paediatric patients in whom targeted dosing with both etoposide and carboplatin was possible using detailed pharmacokinetic sampling over three doses. In this latter study, etoposide exposure was within 14% of the target in all four courses studied, despite etoposide plasma clearances varying between 14 and 23 ml $min^{-1}$ $m^{-2}$.

Adaptive control of etoposide dosing based upon both pharmacokinetic and pharmacodynamic factors has been used by Ratain et al (1989, 1991) to treat patients with small-cell lung cancer. In these latter studies, dosing in the adaptive control arm was based upon the pretreatment white cell count and the plasma etoposide concentration at 24 h, and the pharmacodynamic target was a chosen degree of haematological toxicity. Doses in the conventional dosing arm were calculated according to body surface area. Dose escalation using this approach was possible and patients in the adaptive control arm received a dose that on average was 22% greater than in patients treated without adaptive control.

More recently, Joel et al (1996) have reported the results of a pharmacokinetically based dose escalation study of etoposide given intravenously as a continuous infusion over 5 days. Dose modifications after 18 and 66 h were performed on the basis of plasma etoposide concentrations in order to achieve a target level of 2, 3, 4 or 5 μg $ml^{-1}$. Marked variability was seen in plasma etoposide concentrations before adjustment (27–166% of the target, with only 57% of patients within ± 20% of the target concentration at the 2 μg $ml^{-1}$ level), and this was substantially reduced after adjustment (54–137% of target, with 82% of patients within ± 20% at 66 h. The total etoposide dose per cycle ranged from 200 to 994 mg for the 2 μg $ml^{-1}$ cohort. In comparison, the variability in AUC associated with dosing according to surface area in the present study was between 97% and 129% (Table 3), and in only one patient was an error in excess of 20% seen.

The value of any method for reducing interpatient pharmacokinetic variability depends both on the therapeutic range for the drug, and on the extent of variation present in the population. For most chemotherapeutic drugs, a doubling in administered exposure might be expected to have a significant effect on toxicity or response, and a twofold range in exposure might be an acceptable upper limit. By these criteria, surface area-based dosing would appear to be satisfactory for the relatively homogeneous group of patients studied here, and no benefit from adaptively controlled dosing would be expected.

Furthermore, even if the accuracy of adaptive dosing based upon a limited sampling model could be increased, this would be most unlikely to result in clinically significant improvements, as the pharmacokinetic variability seen after surface area-based dosing is already low. In contrast, patients from our previous series (Lowis et al, 1993), and in many other reported studies, have shown much greater pharmacokinetic variability, and a benefit in these patients might have been expected. It is therefore important to define the population of patients receiving etoposide, identifying those for whom conventional surface area-based dosing is appropriate, and those for whom pharmacokinetically guided dosing may offer some benefit. In the present series of children relatively little interpatient variability was identified in pharmacokinetic parameters. The reasons for this are not clear, although only one patient had received prior cisplatin therapy. No benefit from pharmacokinetically guided dosing was seen in these patients, and further studies are indicated in order to identify those patients for whom improved accuracy may be seen. The feasibility of this study with minimal patient inconvenience has however been demonstrated.

### ACKNOWLEDGEMENTS

The authors are grateful to the patients and parents of the patients who took part in this study. The help of the medical and nursing staff of the Department of Child Health, Royal Victoria Infirmary, Newcastle, is also appreciated. Lastly, the financial support of the North of England Children's Cancer Research Fund and the North of England Cancer Research Campaign is gratefully acknowledged.

### REFERENCES

Aisner J, Van Echo DA, Whitacre M and Wiernik PH (1982) A phase I trial of continuous infusion VP16-213 (Etoposide). *Cancer Chemother Pharmacol* **7**: 157–160

© Cancer Research Campaign 1998

Bennett CL Sinkule JA, Schilsky RL, Senekjan EA and Choi KE (1987) Phase I clinical and pharmacological study of 72 hour continuous infusion of etoposide in patients with advanced cancer. *Cancer Res* **47**: 1952–1956

Boos J, Krumpelmann S, Schulze-Westhoff P, Euting T, Berthold F, Jurgens H (1995) Steady-state levels and bone marrow toxicity of etoposide in children and infants: does etoposide require age-dependent dose calculation? *J Clin Oncol* **13**: 2954–2950

D'Argenio DZ and Schumitzky A (1979) A program package for simulation and parameter estimation in pharmacokinetic systems. *Comput Progr Biomed* **9**: 115–134

English MW, Lowis SP, Peng B, Pearson ADJ, Newell DR, Craft AW (1996) Pharmacokinetically guided dosing of carboplatin and etoposide during peritoneal dialysis and haemodialysis. *Br J Cancer* **73**: 776–780

Fukuoka M, Masuda N, Negoro S, Takada M, Kudoh S, Kusunoki Y, Matsui K, Takifuji N, Tachikawa A and Kawahara M (1991) A phase I study of chronic daily oral etoposide in combination with cisplatin for patients with advanced cancer. Cancer **68**: 284–288

Gentili D, Zucchetti M, Torri V, Sessa C, de Jong J, Cavalli F and D'Incalci M (1993) A limited sampling model for the pharmacokinetics of etoposide given orally. Cancer Chemother Pharmacol **32**: 482–486

Henwood JM and Brogden RN (1990) Etoposide: a review of its pharmacodynamic and pharmacokinetic properties, and therapeutic potential in combination chemotherapy of cancer. *Drugs* **39**: 438–490

Joel SP, Heap L, Robbins S, Clarke PI and Slevin ML (1990) A limited sampling strategy for the calculation of etoposide pharmacokinetics. *Proc Am Soc Clin Oncol* **9**: 67

Joel SP, Ellis P, O'Byrne K, Papamichael D, Hall M, Penson R, Nicholls S, O'Donnell C, Constantinou A, Woodhull J, Nicholson M, Smith I, Talbot D and Slevin M (1996) Therapeutic monitoring of continuous infusion etoposide in small-cell lung cancer. *J Clin Oncol* **14**: 1903–1912

Kunitah H and Watanabe K (1994) Phase I/II and pharmacologic study of long-term continuous infusion etoposide combined with cisplatin in patients with advanced non-small cell lung cancer. *J Clin Oncol* **12**: 83–89

Lokich J and Corkery J (1981) Phase I study of VP-16–213 (etoposid) administered as a continuous 5 day infusion. *Cancer Treat Rep* **65**: 887–889

Lokich J, Anderson N, Bern M, Wallach S, Moore C and Williams D (1989) Etoposide admixed with cisplatin. Phase I clinical investigation of 72 hour infusion. *Cancer* **63**: 818–821

Lowis SP, Pearson ADJ, Newell DR and Cole M (1993) Etoposide pharmacokinetics in children: the development and prospective validation of a dosing equation. *Cancer Res* **53**: 4881–4889

Miller AA and Tolley EA (1994) Predictive performance of a pharmacodynamic model for oral etoposide. *Cancer Res* **54**: 2080–2083

Minami H, Shimokata K, Saka H, Saito H, Ando Y, Senda K, Nomura F and Sakai S (1993) Phase I clinical and pharmacokinetic study of 14-day infusion of etoposide in patients with lung cancer. *J Clin Oncol* **11**: 1602–1608

Pflüger K-H, Schmidt L, Merkel M, Jungclas H and Havermann K (1987) Drug monitoring of etoposide (VP16-213). *Cancer Chemother Pharmacol* **20**: 59–60

Pflüger K-H, Hahn M, Hoz J-B, Schmidt L, Kohl P, Fritsch H-W, Jungclas H and Havermann K (1993) Pharmacokinetics of etoposide: correlation of pharmacokinetic parameters with clinical conditions. *Cancer Chemother Pharmacol* **31**: 350–356

Ratain MJ, Schilsky RL, Choi KE, Guarnieri C, Grimmer D, Vogelzang NJ, Senekjian E and Liebner MA (1989) Adaptive control of etoposide administration: Impact of interpatient pharmacodynamic variability. *Clin Pharmacol Ther* **45**: 226–233

Ratain MJ, Mick R, Schilsky RL, Vogelzang NJ and Berezin F (1991) Pharmacologically based dosing of etoposide: a means of safely increasing dose intensity. *J Clin Oncol* **9**: 1480–1486

Relling MV, McLeod HL, Bowman LC and Santana VM (1994) Etoposide pharmacokinetics and pharmacodynamics after acute and chronic exposure to etoposide. *Clin Pharmacol Ther* **56**: 503–511

Sheiner LB and Beal SL (1981) Some suggestions for measuring predictive performance. *J Pharmacokinetics Biopharmaceutics* **9**: 503–512

Strömgren AS, Sörensen BT, Jakobsen P and Jakobsen A (1993) A limited sampling method for estimation of the etoposide area under the curve. *Cancer Chemother Pharmacol* **32**: 226–230

© Cancer Research Campaign 1998